# Wnt/β-Catenin Signaling Mediated-UCH-L1 Expression in Podocytes of Diabetic Nephropathy

**DOI:** 10.3390/ijms17091404

**Published:** 2016-08-25

**Authors:** Hongxia Zhang, Weili Luo, Yonghong Sun, Yanchun Qiao, Liying Zhang, Zhilian Zhao, Shijun Lv

**Affiliations:** 1 Department of Clinic Pathology, Weifang Medical University, Weifang 261053, China; zhanghx@wfmc.edu.cn (H.Z.); liying7h@163.com (L.Z.); 2 Division of Nephrology, Zhongshan Hospital, Fudan University, Shanghai 200032, China; luoweililuoweili@163.com; 3 Department of Pathology, Affiliated Hospital of Weifang Medical University, Weifang 261031, China; sunyonghong88@163.com (Y.S.); yan7902@163.com (Y.Q.); 4 Department of Pathology, Maternal and Child Care Service Centre of Changyi, Changyi 261300, China; 15963622065@163.com

**Keywords:** podocytes, UCH-L1, Wnt/β-catenin, DN

## Abstract

Increasing studies identified podocyte injury as a key early risk factor resulting in diabetic nephropathy (DN). The ubiquitin carboxy-terminal hydrolase 1 (UCH-L1) participates in podocyte differentiation and injury, which is elevated in the podocytes of a variety of nephritis. Whether UCH-L1 expression is positively related to podocyte injury of DN remains unclear. In this study, elevated expression of UCH-L1 and its intrinsic mechanism in high glucose (HG)-stimulated murine podocytes were investigated using western blot and real-time quantitative PCR. Kidney biopsies of DN patients and health individuals were stained by immunofluorescence (IF) method. The morphological and functional changes of podocytes were tested by F-actin staining and cell migration assay. Results demonstrated that HG induced upregulation of UCH-L1 and activation of the Wnt/β-catenin signaling pathway in podocytes. However, blocking of the Wnt pathway by dickkopf related protein 1 (DKK1) eliminated the above changes. Furthermore, IF staining confirmed that, compared with healthy individuals, the expression of UCH-L1 and β-catenin were obviously increased in kidney biopsy of DN patients. Overexpression of UCH-L1 remodeled its actin cytoskeleton, increased its cell migration and impacted its important proteins. All the findings manifested that Wnt/β-catenin/UCH-L1 may be a new potential therapy method in the treatment of DN in future.

## 1. Introduction

Diabetic nephropathy (DN) is a major complication of diabetes mellitus (DM). The detectable early marker of DN is the appearance of microalbuminuria, followed by proteinuria, hyperfiltration, and up to reductions in the glomerular filtration rate, which manifests the destruction of the glomerular filtration barrier (GFB). Podocytes are terminally differentiated epithelial cells locating in the inner layer of Bowman’s capsule that constitute the outer layer of GFB. Their normal structure and function play a major role in maintaining normal integrity of the GFB, which explain why podocyte injury is generally associated with increasing obvious proteinuria [1]. HG contributes to the gradually damage of GFB and a progressive proteinuria, leading to end-stage renal disease (ESRD) [2]. However, the mechanisms of podocyte injury induced by HG are not well explored.

The ubiquitin carboxy-terminal hydrolase 1 (UCH-L1, PGP9.5) which is an important member of the deubiquitinating enzymes family constituting the ubiquitin-proteasome system (UPS), plays an important role in the regulation of many proteins [3,4]. It was first mainly found in brain and testis. Furthermore, it is also expressed in kidney and involves in nephrogenesis [5,6,7]. Our previous work and other lab had reported that UCH-L1 is involved in podocyte differentiation and injury. It is upregulated in the podocytes of several forms of immune complex-mediated nephritis [8,9,10]. Increasing studies identified podocyte injury as a vital early risk factor resulting in DN [11,12]. Whether UCH-L1 expression is related to podocyte injury of DN is still not known.

The Wnt/β-catenin pathway, so-called canonical Wnt pathway, is activated through binding of Wnt ligand proteins to members of the Frizzled receptor protein family. Then such an interaction inhibits the degradation of β-catenin, followed by its translocation into the nucleus. In the nucleus, β-catenin subsequently initiates the combining with the DNA-bound T cell factor/lymphocyte enhancement factor (TCF/LEF) of many different transcription factors and then triggering transcriptional responses of a variety of Wnt target genes involved in cellular survival, proliferation and differentiation [13]. The Wnt/β-catenin pathway plays an important part in a variety of kidney diseases, including DN [14]. Li et al. reported that the classic Wnt/β-catenin pathway modulated TRPC6 protein expression and provoked the subsequent podocyte injuries in HG-treated podocytes [15]. Duan et al. proved that Wnt1, p-GSK-3β/tGSK-3β, p-β-catenin/tβ-catenin were higher in the DN group mice than control and the rhein therapy ameliorated kidney injury through wnt/β-catenin signaling in DN mice [16]. Hideki Kato found mice with podocyte-specific expression/deletion of stabilized Ctnnb1 showed increased susceptibility to DKD respectively and emphasized that balanced β-catenin expression is critical for the maintenance of GFB [17]. Therefore, whether UCH-L1 expression is increased or not in HG-stimulated podocytes and is mediated by Wnt/β-catenin is not clear at present.

Therefore, the present study was implemented to determine whether high glucose induces podocyte injury through upregulation of UCH-L1 and to explore the possible mechanisms.

## 2. Results

### 2.1. High Glucose Increased the Expression of UCH-L1 in Podocytes in Vitro

**Figure 1:**
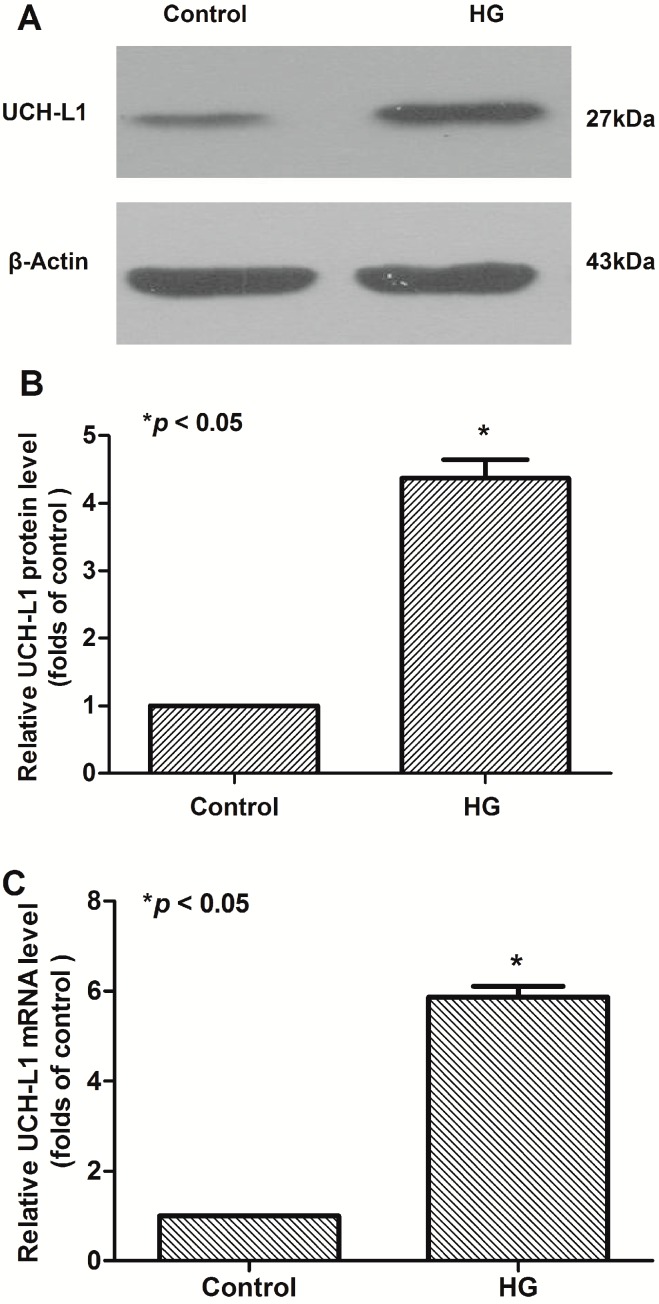
UCH-L1 expression induced by high glucose in podocytes. Podocytes were maintained or not in high glucose (30 mM) for 48 h. Podocyte lysate proteins (60 μg) were analyzed respectively by western blot using murine anti-UCH-L1 antibody and β-Actin was used as control for protein loading. (**A**) Western blot assay of UCH-L1; (**B**) corresponding statistic histogram of UCH-L1 protein expression; (**C**) quantitative real-time RT-PCR assay of UCH-L1 mRNA. Data representative of three independent experiments. * *p* < 0.05 compared to control group.

The cultured podocytes were divided into the control group and the HG group which were treated with HG for 48 h. The expression of UCH-L1 in the HG group was significantly higher than that in the control one (Figure 1A,B). At the mRNA level, quantitative real time RT-PCR showed the increase of UCH-L1 in HG-treated cells were greater than that in the control group, ~4.4-fold for 48 h (Figure 1C). Overall, these results indicated that HG upregulated the expression of UCH-L1 in podocytes at both the mRNA and protein levels.

### 2.2. Activation of Wnt/β-Catenin Pathway Induced by High Glucose in Podocytes

**Figure 2:**
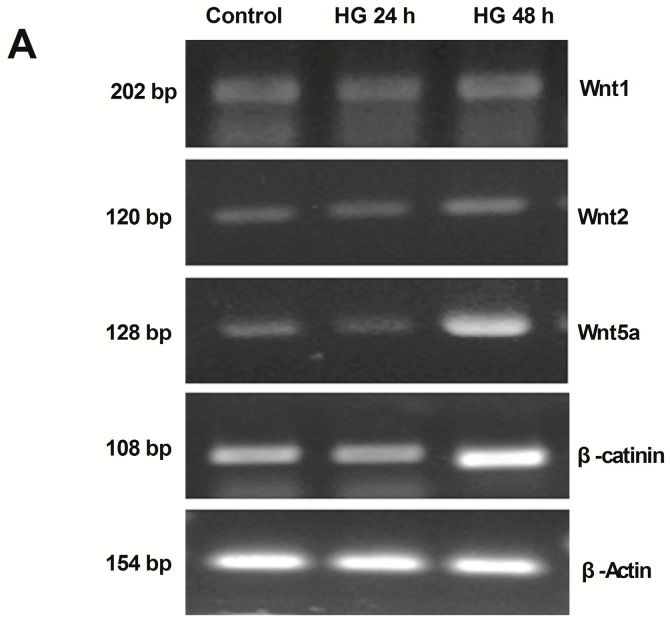

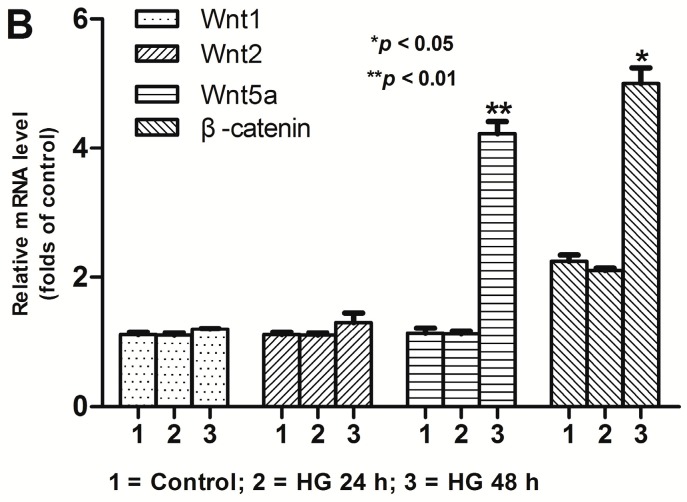
Activation of the Wnt/β-catenin pathway induced by high glucose in podocytes Podocytes incubated with HG for 24 and 48 h, respectively. (**A**) Reverse transcription-PCR (RT-PCR) demonstrating an non-altered expression of Wnt1 and Wnt2 and an increased expression of Wnt5a and β-catenin compared to control; (**B**) corresponding statistic histogram of Wnt1, Wnt2, Wnt5a and β-catenin. Data representative of three independent experiments. * *p* < 0.05, ** *p* < 0.01 compared to control group and HG 24 h group respectively.

An analysis of the mRNA expression of common measured Wnt genes was detected by reverse transcription-PCR (RT-PCR) approach to explore the activation status of Wnt/β-catenin signaling pathway in the control group and the HG group. As shown in Figure 2, compared to control, mRNA expression of Wnt1 and Wnt2 were not markedly changed in the HG group, neither by 24 h nor by 48 h. However, after HG treatment, mRNA expression of Wnt5a was evidently increased by 48 h, ~3.1 folds.

To examine the biological consequence of Wnt induction in HG induced podocyte injury, the mRNA expression of β-catenin, the principal downstream mediator of the canonical Wnt signaling, was also investigated in podocytes treated with HG. As shown in Figure 2, RT-PCR approach revealed a marked induction of β-catenin mRNA expression in podocytes by 48 h after HG treatment. All these results suggested that the canonical Wnt pathway might play a key role in HG-treated podocytes.

### 2.3. Inhibition of Wnt/β-Catenin Pathway Downregulated High Glucose-Induced UCH-L1 Upregulation

**Figure 3:**
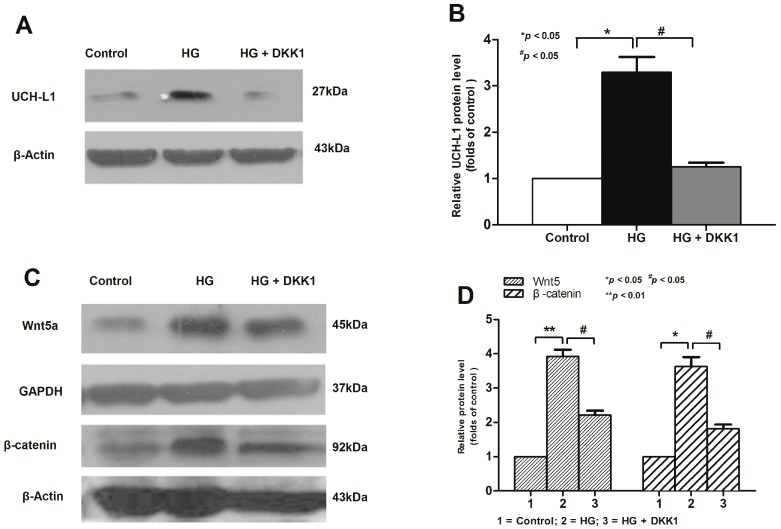
Inhibition of the Wnt/β-catenin pathway downregulated HG-induced UCH-L1 expression. Podocytes were stimulated with HG or HG + DKK1 (200 ng/mL) for 48 h. (**A**) Western blot assay of UCH-L1 protein level; (**B**) corresponding statistic histogram of UCH-L1 protein expression; (**C**) western blot assay of Wnt5a and β-catenin protein level; (**D**) corresponding statistic histogram of Wnt5a and β-catenin expression. Data representative of three independent experiments. * *p* < 0.05, ** *p* < 0.01 compared to control group. ^#^ *p* < 0.05 compared to HG + DKK1 group.

In the transformed cells, β-catenin can upregulate the expression of endogenous UCH-L1 mRNA and protein [18]. However, it is unknown whether the canonical Wnt/β-catenin signaling mediates high glucose induced upregulation of UCH-L1 in podocytes. To provide the intrinsic mechanism of UCH-L1 upregulation in HG-stimulated podocytes, podocyte cells were treated with HG for 24 or 48 h, after preincubated with or without recombinant Dickkopf-1 (DKK1) protein, a secreted Wnt antagonist that can specially block the canonical Wnt signaling [19]. As shown in Figure 3A,B, preincubation with DKK1 obviously reduced UCH-L1 expression in HG-stimulated podocytes at the protein levels by 48 h. Meanwhile, western blot results demonstrated that the expression of Wnt5a and β-catenin were obviously elevated in HG-stimulated podocytes. However, preincubation with DKK1 significantly lowed the expression of the aforementioned two proteins (Figure 3C,D), which confirmed that the canonical Wnt/β-catenin pathway was really blocked and such blockage reduced UCH-L1. All the above results suggested an important upstream role for Wnt/β-catenin in mediating the upregulation of UCH-L1 in HG environment.

### 2.4. UCH-L1 and β-Catenin Distribution in Podocytes of Diabetic Nephropathy Patients

**Figure 4:**
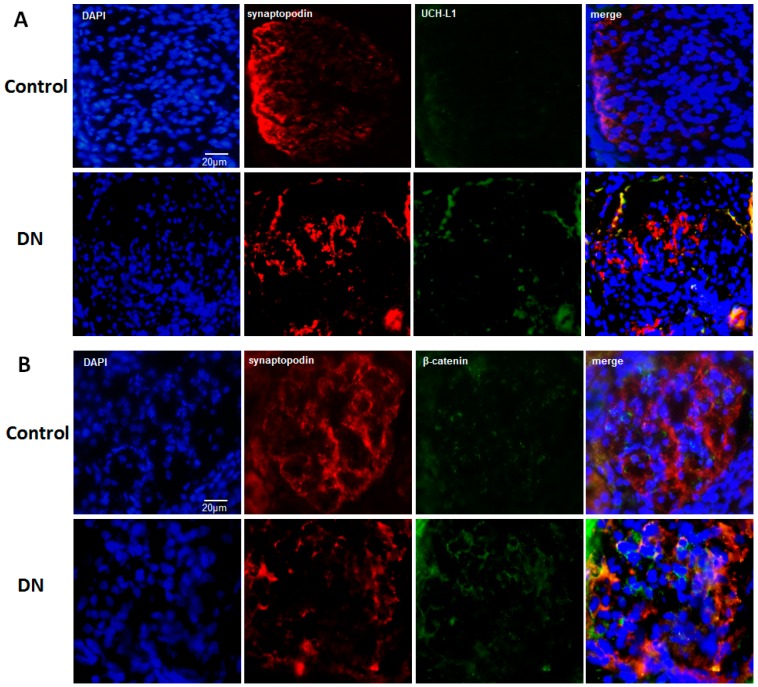
(**A**) UCH-L1 expression in podocytes of diabetic nephropathy patients. Immunofluorescence staining was performed in 4-μm-thick frozen sections from healthy control persons and DN patients. Synaptopodin showed in red, UCH-L1 showed in green and DAPI showed in blue (*n* = 6). Original magnification, 200×; (**B**) β-catenin expression in podocytes of diabetic nephropathy patients. Immunofluorescence staining was performed in 4-μm-thick frozen sections from healthy controls persons and DN patients. Synaptopodin showed in red, β-catenin showed in green and DAPI showed in blue (*n* = 6). Original magnification, 200×.

To investigate the potential role of Wnt/β-catenin/UCH-L1 signaling in podocyte injury in vivo, immunofluorescence staining was used to explore the expression of UCH-L1 and β-catenin in podocytes of diabetic nephropathy patients. We selected typical DN slides to further assess. The typical images of DN were that parts of glomeruli experienced nodular sclerosis, together with increase of mesangial matrix, appearance of fibrin cap and thickening of capillary wall. The healthy control slides had none of the above features. As shown in Figure 4A, double immunofluorescence staining for both synaptopodin (red) and UCH-L1 (green) revealed that UCH-L1 can be evidently positioned in the podocytes (marked by synaptopodin) of diabetic nephropathy patients. Positive staining of UCH-L1 was mainly located at the peripheral area of the capillary tufts in accord with the distribution of synaptopodin. However, in the healthy control slides, there was no obvious positive staining of UCH-L1. As for β-catenin, which was shown in Figure 4B, weak staining was detectable in the healthy control slides, however, strong positive staining was detectable in synaptopodin-positive staining podocytes in diabetic nephropathy patients. These results suggested that in podocytes of diabetic nephropathy, both UCH-L1 and β-catenin were increased, which exhibited consistent results with those of cells experiments in vivo.

### 2.5. UCH-L1 Overexpression Induced Changes of F-Actin and Hypermotility in Podocytes

**Figure 5:**
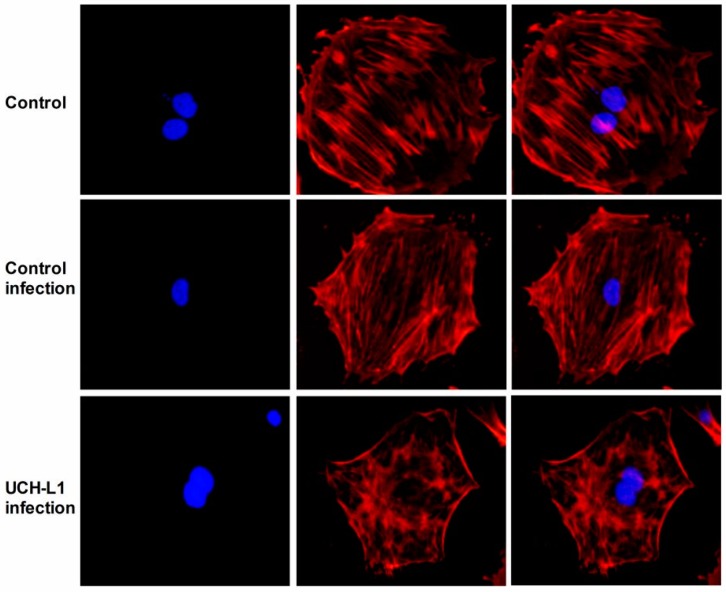
Effect of UCH-L1 overexpression on structure injury in podocytes. Immunofluorescence staining of F-actin was performed in podocytes of control group, control infection group and UCH-L1 infection group. F-actin was showed in red, cell nucleus showed with DAPI in blue (*n* = 5). Original magnification, 200×.

To investigate the effect of increased UCH-L1 to podocyte structure in vivo, cytoskeleton was stained with F-actin staining in UCH-L1 adenoviral vector-infected podocytes and control group and control infection group. As the results shown in Figure 5, in both control groups podocytes displayed an obvious multiple-process morphological structure, and all the actin fibers arranged in order as nearly paralleled fasciculate models in the cytoplasm. In contrast, in UCH-L1 adenoviral vector-infected podocytes, the morphology of podocytes significantly changed. The thin process became less and the actin fibers became out-of-order and intertwined with each other, which were mostly around the nucleus. These results indicated that UCH-L1 overexpression could change podocytes morphological structure.

**Figure 6:**
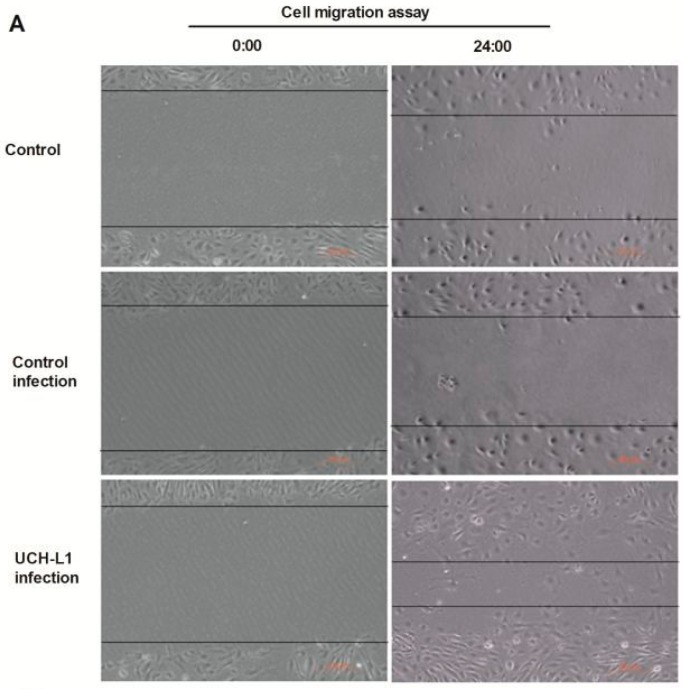

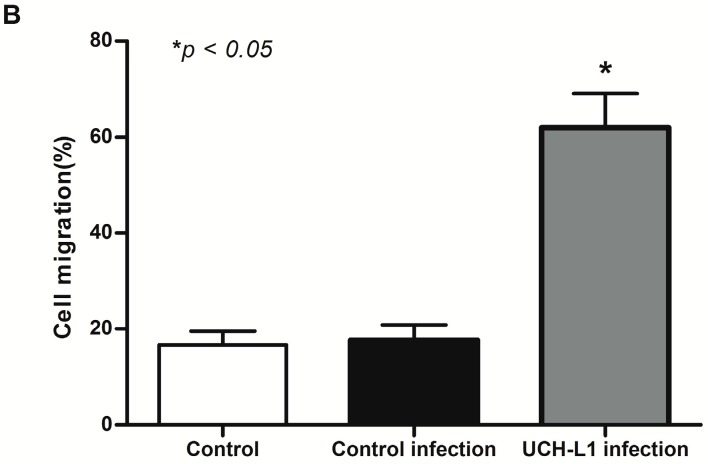
UCH-L1 overexpression induced podocyte hypermotility as assessed by cell migration assay. (**A**) Differentiated podocytes were infected with UCH-L1 overexpression adenoviral vector. Control cells were treated with vehicle vector or were not treated. Then subsequently scratch was processed using a 0.1 mL pipette. The observation was made immediately after scratch (0:00 h) and at 24:00 h, Original magnification, 200×; (**B**) quantification of the cell migration area by computerized morphometric analysis. Data are given as means + SD. *n* = 20 areas from three independent experiments. * *p* < 0.05 versus control group and control infection group, respectively.

Further, a cell migration assay was carried to assess the motility of podocytes in UCH-L1 adenoviral vector-infected podocytes and control groups. As shown in Figure 6, compared with both control groups, UCH-L1 infection could significantly prompt the motility of podocytes, lessen the distances between the leading edges of the migrating podocytes. The results suggested that UCH-L1 overexpression may empower hypermotility of podocytes. Combining the above results, it was clear that UCH-L1 overexpression could affect the morphological and functional changes of podocytes.

### 2.6. UCH-L1 Overexpression Changed the Expression of Several Proteins in Podocytes

**Figure 7:**
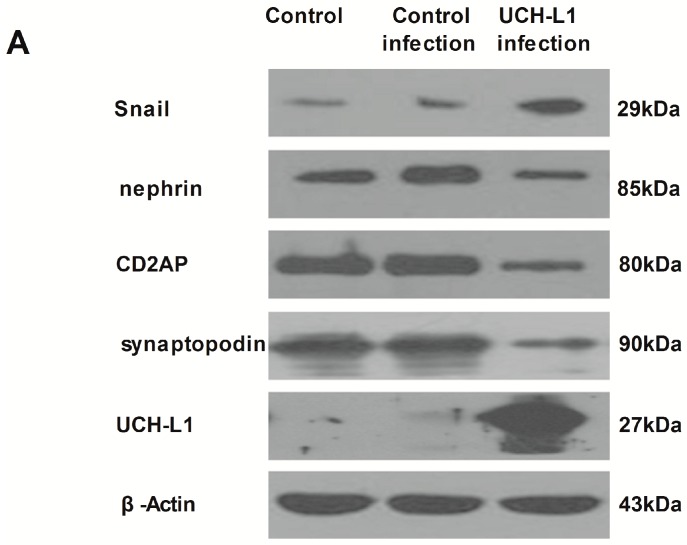

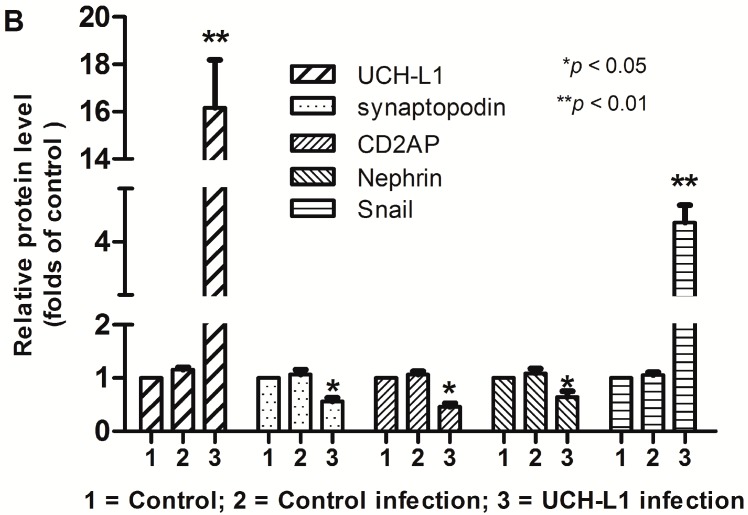
UCH-L1 overexpression induced changes of expression of several proteins in podocytes. Differentiated podocytes were infected with UCH-L1 overexpression adenoviral vector for 48 h. Control cells were treated with vehicle vector or were not treated for 48 h. Podocytes lysate proteins (60 μg) were analyzed respectively by western blot using murine corresponding antibodies and β-Actin was used as control for protein loading. (**A**) Western blot assay of UCH-L1, synaptopodin, CD2AP, nephrin and Snail; (**B**) corresponding statistic histogram of UCH-L1, synaptopodin, CD2AP, nephrin, and Snail protein expression. Data representative of three independent experiments. * *p* < 0.05, ** *p* < 0.01 both versus control group and control infection group.

To further investigate the effect of increased UCH-L1 in podocytes, several important molecules of podocytes were assessed after differentiated podocytes were infected with UCH-L1 overexpression adenoviral vector for 48 h. As shown in Figure 7, expression of UCH-L1 in UCH-L1 infected-group was significantly high than that of control group and control infection group, which demonstrated the successful infection. The successful infection decreased the expression of synaptopodin, CD2AP and nephrin and increased that of Snail. These results demonstrated that UCH-L1 could change the expression of important proteins in podocytes, which may correspondingly affect its structure and function.

## 3. Discussion

**Figure 8:**
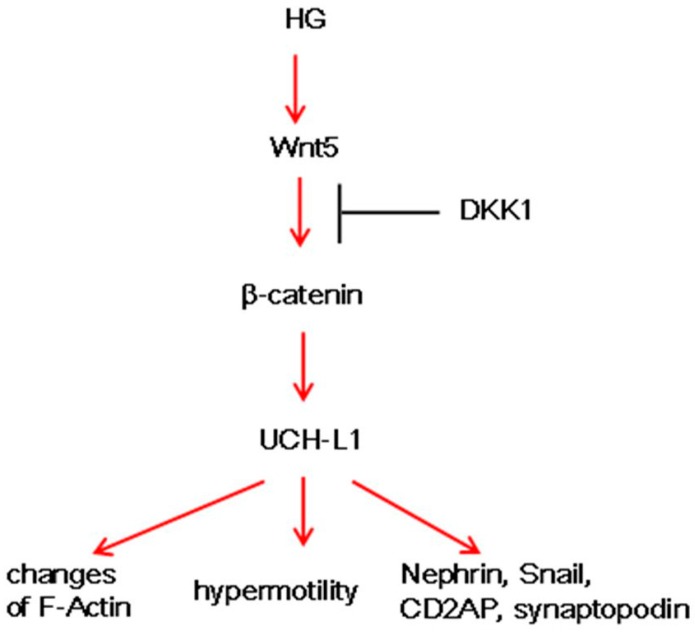
Diagram shows the Wnt/β-catenin/UCH-L1 signaling leading to podocyte injury in HG environment. HG induces Wnt5, activates β-catenin, and elevates UCH-L1, followed by changes of F-actin, hypermotility and changes of some proteins, which can be blocked by DKK1.

In this study, results demonstrated that HG upregulated the expression of UCH-L1 in murine podocytes, which might be mediated by the Wnt/β-catenin signaling pathway. Furthermore, IF results proved such close relationship between β-catenin and UCH-L1 in DN patients. The overexpression of UCH-L1 not only changed F-actin and hypermotility of podocytes but also changed several important proteins (Figure 8). All the above findings illustrated that the Wnt/UCH-L1 may present a new injury mechanism of DN.

Our earlier work had proved that UCH-L1 is expressed aberrantly in the injured podocytes of many nephrites, especially the immunocomplex-mediated nephrites [20,21]. Podocyte injuries are closely related to DN [22,23]. Whether UCH-L1 expression is increased in podocytes of DN has not been known. In this experiment, findings demonstrated that HG significantly upregulated UCH-L1 in mouse podocyte cells (Figure 1, *p* < 0.05). Meanwhile, UCH-L1 was obviously detected in the kidney biopsy of the DN patients (Figure 4A). Experiments in vivo and in vitro implicated that the expression of UCH-L1 is abnormally elevated in high glucose environment.

Then, the possible mechanism of UCH-L1 expression was explored. The Wnt/β-catenin pathway is involved in cellular growth and differentiation in many diseases, such as DN and IgA nephropathy [13,14]. In our study, the results indicated that Wnt5a, one number of the Wnt protein family, and β-catenin, the common downstream mediator of Wnt/β-catenin signaling, were activated in HG-stimulated podocytes (Figure 2), which confirmed the other studies. In addition, it should be noted that it was Wnt5 but not Wnt1 and Wnt2 that was induced, indicating different members were selectively activated in different circumstances. Blockading the Wnt/β-catenin pathway by DKK1 could low the expression of Wnt5a and β-catenin, followed by down-regulation of UCH-L1 at protein levels (Figure 3). The above results demonstrated the Wnt/β-catenin signaling pathway mediated the UCH-L1 expression in high glucose-stimulated podocytes. Furthermore, IF experiment results also illustrated that in kidney biopsy of DN patients, both β-catenin and UCH-L1 are more significantly detected than those of control health persons (Figure 4), which further proved the close relationship between Wnt/β-catenin pathway and UCH-L1.

UCH-L1 plays a vital role in regulating a broad variety of membrane proximal events, such as, cytoskeleton rearrangement, cell adhesion and motility. UCH-L1 endowed pathogenic bacteria with receptor clustering, remodeling of the actin cytoskeleton, and subsequently invading epithelial cells. Furthermore, UCH-L1 was sufficient to promote formation of actin stress fibers [24]. UCH-L1 could promote invasive capacity of malignant B cells through inhibiting LFA-1 dependent homotypic adhesion [25]. UCH-L1 facilitated HeLa cells migration and survival, inhibited anoikis, and boosted anchorage independent growth [26]. All the studies demonstrated that UCH-L1 could regulate actin cytoskeleton and cell migration. In DN, activation of several signaling pathways can cause podocyte foot processes fusion or even deletion [22,23]. On the premise that HG increased the expression of UCH-L1, we continued to investigate the influence of UCH-L1 overexpression on podocyte morphology and cell migration. After infected by UCH-L1 adenoviral vector, podocytes appeared that the thin process became less and the intracellular actin fibers became out-of-order and intertwined with each other, which were mostly around the nucleus (Figure 5). Furthermore, the cell migration assay proved that podocytes infected by UCH-L1 adenoviral vector, compared with the control groups, had an obviously increased capability of motility (Figure 6).

In addition, a following experiment was conducted to test the impact of UCH-L1 overexpression on important podocytes proteins. As illustrated in our early study [21], UCH-L1 overexpression could decrease the expression of nephrin, a slit diaphragm protein that is a hallmark for podocyte injury, and increased the expression of Snail, a special transcription factor that is involved in mediating podocyte morphology and function (Figure 7). Beyond that, UCH-L1 overexpression lowered the expression of synaptopodin and CD2AP (Figure 7). Synaptopodin, which is the major constituent protein of the podocyte cytoskeleton and can stabilize GFB, primarily located in the terminal processes of podocytes. CD2AP in podocytes serves as an adaptor protein which can bridge nephrin to podocin, anchor these slit diaphragm proteins to podocyte actin filaments and send signals inward or outward. There is a close correlation between the two proteins and podocyte cytoskeleton. Maybe, in HG circumstance, the increased UCH-L1 which was mediated by Wnt/β-catenin pathway changed several important proteins of podocytes, and corresponding remolded actin cytoskeleton and improved cell migration. However, in our study, we have not clearly explored how UCH-L1 changed such important proteins, which deserves further study.

## 4. Materials and Methods

### 4.1. Cell Culture

The conditionally thermosensitive SV40-transfected immortalized murine podocyte cell line MPC5 (a gift from Xueguang Liu, College of Basic Medicine of Fudan University, Shanghai, China) was cultured as described [21]. In brief, cells were cultured on collagen I-coated (Sigma-Aldrich, St. Louis, MO, USA) plates under permissive conditions (33 °C, 5% (*v*/*v*) CO2, RPMI 1640, 10% (*v*/*v*) fetal bovine serum, 50–10 U/mL γ-interferon). Podocytes between passages 5 and 20 were used for the proposed experiments. Then cells were cultured under nonpermissive conditions (37 °C without γ-interferon) for 10–14 days for differentiation. After differentiation, podocytes were divided into many groups and received different treatments, including high concentration of d-glucose (30 mM) , dickkopf related protein 1 (DKK1) (R & D System Inc., Minneapolis, MN, USA) or adenoviral vector infection (constructed by Hanbio Biotechnology Co., Ltd., Shanghai, China) according to the manufacturer’s instructions.

### 4.2. Western Blot Analysis

Western blot analysis was done as described. In brief, portions (15 μL) of the podocyte lysates including soluble cell proteins were separated by SDS-PAGE (10% (*w/v*) polyacrylamide gel), transferred onto a PVDF membrane (Millipore, Eschborn, Germany) and then blocked with 5% (*v*/*v*) nonfat dried milk in Tris buffered saline Tween (TBST, 20 mM Tris, pH 7.6, 137 mM NaCl, and 0.1% Tween 20). Then, the membrane was probed with primary antibodies against UCH-L1 (1:2000, Millipore), synaptopodin (1:1000, Gene Tex, Irvine, CA, USA), CD2-associated protein (CD2AP) (1:2000, CST, Danvers, MA, USA), nephrin (1:2000, Abcam, Cambridge, UK) , Snail (1:500, Abcam), Wnt5a (1:1000, CST), β-catenin (1:2000, CST) , β-Actin (1:2000, Proteintech Group, Rosemont, IL, USA) and GAPDH (1:2000, Proteintech Group, Rosemont, IL, USA) overnight at 4 °C followed by incubation with horseradish peroxidase -conjugated secondary antibody (1:10,000, Protein Tech Group). The immunoreactive bands were visualized by ECL (Pierce Biotechnology, Rockford, IL, USA) and visualized on Kodak X-ray films. Each experiment was done at least three times. Densitometry analysis of the images obtained from X-ray films was performed using the Image J software (NIH, Bethesda, MD, USA).

### 4.3. Quantitative Real-Time RT-PCR

Total RNA was extracted from cells (2 × 10^6^) using TRIzol^®^ reagent (Sangon, Shanghai, China) according to the manufacturer’s instructions. Then, total RNA (500 ng/reaction) was reverse transcribed (RT) into cDNA using Transcriptor First Strand cDNA Synthesis Kit (Roche, Mannheim, Germany). RT-PCR was done with FastStart Taq DNA polymerase (Roche, Mannheim, Germany). The productions of RT-PCR were run on agarose gel. Real-time RT-PCR was done with LightCycler^®^ FastStart DNA Master SYBR Green I (Roche) (primer sequences are given in Table 1). The mRNA levels of target genes were calculated following normalization to the β-Actin mRNA levels by the comparative delta threshold cycle (∆∆C_t_) method. All reactions consisted of 39 cycles of 5 s at 94 °C, 30 s at 60 °C, 20 s at 72 °C. The specificity of the amplification reactions was confirmed by melt curve analysis.

### 4.4. Immunofluorescence

DN tissues from renal needle biopsies and the control healthy kidney tissue from the normal kidney tissue around the kidney tumor were collected from Department of Pathology, affiliated hospital of Weifang Medical University, China within 2014 in accordance with local ethical guidelines. Permission to use the tissues for research purposes was obtained and approved by the Ethics Committee from Weifang Medical University, China, and a written consent form was obtained from all patients and control heath individuals. All the patients suffered from severely proteinuria (≥3.5 g/24 h) and their fasting glucose levels were more than ≥7.0 mmol/L. Kidney slices at 4 mm thickness were fixed for 15 min in 4% paraformaldehyde, followed by permeabilization with 0.2%. Triton X-100 in PBS for 10 min at room temperature. After blocking with 10% goat serum for 60 min, slides were immunostained with primary antibodies against UCH-L1 (1:100), β-catenin (1:100, CST) and synaptopodin (1:100). To visualize the primary antibodies, slices were stained with FITC- or TRITC-conjugated secondary antibodies (Proteintech Group). The slices were viewed under a fluorescence microscope (Olympus, Tokyo, Japan).

### 4.5. Cell Migration Assay

Podocytes were seeded in the 6-well plate and infected with or without UCH-L1 overexpression adenoviral vector. Then the confluent monolayers podocytes were scraped with a 0.1 mL pipette. Images of the same area were acquired at indicated time points using an inverted microscope and were analyzed using the Image J software image processing program. The percentage of cell migration area was calculated as (Area_0 h_ − Area_indicated time_)/Area_0 h_.

### 4.6. F-Actin Staining

Podocytes were seeded on sterile cover slips in the 6-well plate and infected with or without UCH-L1 overexpression adenoviral vector for 48 h. After washing with PBS, the podocytes were fixed in 4% paraformaldehyde for 10 min, then permeabilized with 0.1% Triton X-100-PBS for 15 min, and blocked with 3% bovine serum albumin for 30 min at room temperature. F-actin was stained with phalloidin eFluor^®^ 570 (eBioscience, San Diego, CA, USA) for 30 min at room temperature. After being countmounted with DAPI-containing mounting solution, the slides were examined with a fluorescence microscope (Olympus). Image J software was used for post-processing of the images, including merging and colocalization analysis.

### 4.7. Statistical Analysis

All data analysis was done with SPSS software (SPSS, Chicago, IL, USA) and was given as mean ± SEM. Paired means were analyzed using Student’s *t*-test. Multiple means more than 2 groups were analyzed using ANOVA. Statistically significant difference was set at *p* < 0.05 and highly significant difference was set at *p* <0.01. All experiments were performed at least in triplicate.

## 5. Conclusions

Our data demonstrated that HG could activate the canonical Wnt/β-catenin signaling pathway and then enforce the expression of UCH-L1 in high glucose-treated murine podocytes at the protein and mRNA level, which is confirmed in kidney biopsy of DN patients by IF. The increased UCH-L1 can induce morphological and functional changes of podocyte cells. All these results suggested that Wnt/β-catenin/UCH-L1 might be a new potential therapy method in the treatment of DN.

## Figures and Tables

**Table 1:** Nucleotide sequences of the primers used for RT-PCR in this study.

Mouse Gene	Primer Sequence 5′ to 3′	
	Forward	Reverse
*UCH-L1*	AAGCAGACCATCGGAAACTC	GCTCTATCTTCGGGGGACA
*Wnt1*	TGACCTCTTTGGGTATTATCA	GAGACAAGGAGAATGTAGGGT
*Wnt2*	CTCGGTGGAATCTGGCTCTG	CACATTGTCACACATCACCCT
*Wnt5a*	CAACTGGCAGGACTTTCTCAA	CATCTCCGATGCCGGAACT
β-catenin	ATGGAGCCGGACAGAAAAGC	CTTGCCACTCAGGGAAGGA
β-Actin	CATCCGTAAAGACCTCTATGCCAAC	ATGGAGCCACCGATCCACA
